# Biogenic and chemically synthesized *Solanum tuberosum* peel–silver nanoparticle hybrid for the ultrasonic aided adsorption of bromophenol blue dye

**DOI:** 10.1038/s41598-020-74254-y

**Published:** 2020-10-13

**Authors:** Kovo G. Akpomie, Jeanet Conradie

**Affiliations:** 1 Physical Chemistry Research Laboratory, Department of Chemistry, University of the Free State, Bloemfontein, South Africa; 2 Industrial/Physical Chemistry Unit, Department of Pure and Industrial Chemistry, University of Nigeria, Nsukka, Nigeria

**Keywords:** Environmental sciences, Chemistry, Engineering, Materials science, Nanoscience and technology

## Abstract

This work was aimed at the synthesis of a hybrid (STpe-AgNP), obtained by impregnation of silver nanoparticles (AgNP) onto *Solanum tuberosum* peel (STpe), for the ultrasonic assisted adsorption of bromophenol blue (BB) dye. SEM, FTIR, XRD, EDX, TGA and BET techniques were used to characterize the adsorbents. The XRD, SEM and EDX confirmed successful impregnation of AgNPs onto STpe to form the hybrid. The AgNPs impregnated onto the hybrid were found to be water stable at various pH values of 2.0–9.0. Chi-square (χ^2^ < 0.024) and linear regression (R^2^ > 0.996) showed that the Freundlich model was best fitted among the isotherm models, corroborated by the oriented site model. Kinetic analysis conformed to the intraparticle diffusion and pseudo-first-order rate equations, while thermodynamics displayed a physical, spontaneous and endothermic adsorption process. The presence of competing Pb(II), Ni(II), Cd(II) and Zn(II) metal ions in solution interfered with the adsorption of BB onto the biosorbents. In terms of reusability, STpe and STpe-AgNP showed BB desorption of 91.3% and 88.5% respectively, using NaOH as eluent. Ultra-sonication significantly enhanced the adsorption of BB by both adsorbents, but the impregnation of AgNPs only slightly improved adsorption of the dye from the simulated wastewater. This study also illustrated that pristine STpe biomass waste is a cheap viable option for the decontamination of BB from water.

## Introduction

The problem of water pollution by contaminants released from industries into the environment is of global concern, due to rapid growth of industries around the world. Dye pollution by effluents from the paper, cosmetic, food, dyeing, leather and textile industries has received serious attention ever since dyes were identified as dangerous water contaminants^1,2^. Dyes are classified among the top three pollutants globally, with over 700,000 tons of dyes consumed by the textile industry alone^3^. They are also known to be hazardous to all living things, negatively affecting photosynthesis in aquatic organisms, as well as being carcinogenic, mutagenic and allergenic^4,5^. Due to their complex chemical structures they are stable, and therefore exhibit resistance to biodegradation, which hinder their removal from water bodies^6^. Bromophenol blue (BB), a triphenylmethane dye, and its related compounds (xanthenes and fluoresceins) are widely applied in laboratory indicators, printing inks, textiles, cosmetics, drugs and food industries^7,8^. The BB dye is also associated with all the negative effects resulting from dye contamination mentioned above^9–11^. Most studies on dye removal have focused on dyes such as methylene blue, congo red, malachite green, methyl orange, rhodamine B, crystal violet and acid yellow, with only few studies on BB, despite its extensive use in industry^12–14^. Therefore, the removal of BB dye from solution should also be given significant attention by researchers.

Several abstraction methods have been utilized for dye removal from solution, such as membrane process, adsorption, precipitation, filtration, electrocoagulation, ozonation, flocculation, biological treatment, floatation, solvent extraction, oxidation, ion exchange, ultraviolent irradiation and photo-degradation^15–18^. Most of these techniques are expensive or ineffective for dye removal^4^. Considered to be the most preferred is the adsorption technique, due to the ease of operation, high effectiveness and design simplicity^19,20^. In addition, activated carbon is widely used and commercially applied for dye decontamination, due to its impressive adsorption capacity^21^, but the high cost is a big limitation for its application^22–24^. The utilization of low-cost materials as suitable alternatives, such as biowastes^25^, chitosan based materials^26^, and natural inorganic materials^27,28^ has gained attention recently. However, biomass waste materials are the preferred choice, owing to their abundance, low cost, biodegradability and efficiency^29,30^.

Potato (*Solanum tuberosum*) peel biomass waste has proved to have great potential for decontamination of dyes as a low-cost waste material with high removal efficiency^31–33^. However, a thorough literature search showed that *Solanum tuberosum* peel (STpe) has not yet been applied for BB sequestration from solution. Additionally, recent research trends have pointed to the use of nano-based materials such as metallic nanoparticles for adsorption or catalytic degradation of dyes from water, which have been found to be highly effective^34–40^. This is attributed to their high selectivity, efficient active sites, high surface area to mass ratio, high adsorption capacity and catalytic activity^41,42^. However, due to the difficulty in separating the metallic nanoparticles from solution, instead the impregnation of these nanoparticles onto suitable adsorbent substrate to form hybrid materials has gained significant attention recently^43,44^. This impregnation of metallic nanoparticles onto adsorbents has been found to enhance the trapping of contaminants from water^45,46^. Our recent study showed that the impregnation of magnetite nanoparticle on *Solanum tuberosum* peel was effective in the biosorption of celestine blue dye^33^. The advantage of such hybrids is that they exhibit improved surface characteristics, due to a combination of the positive properties of both the nanoparticles and the support material making up the composite.

Silver nanoparticle (AgNP) hybrids with clay, zeolite, alumina and activated carbons have been discovered to be highly effective for decontamination of dye and other contaminants from waste solution^47–54^. However, little is known on the application of biomass-AgNPs hybrids for pollutant removal, despite the advantages of utilizing waste biomass materials for adsorption purposes^55^. We therefore prepared a novel material: *Solanum tuberosum* peel**–**silver nanoparticle (STpe-AgNP) hybrid and tested its application for sequestration of BB from solution as a viable efficient adsorbent. We used the major potato species available worldwide, *Solanum tuberosum*, as biosorbent. Furthermore, the application of ultrasonic radiation (sonication) to adsorption has been found to enhance the removal process of the contaminant onto the adsorbent, as a result of agitation and activation of the aggregated particle sites by applying sound energy. Sonication also provides suitable energy to overcome hindrances from mass transfer between adsorbate and adsorbent, leading to efficient interaction^56,57^. Ultrasonic assisted adsorption of BB was therefore applied in this study in an attempt to enhance the adsorption capacity of the STpe-AgNP hybrid for dye removal. The effect of various processing factors, thermodynamics, kinetics, isotherms, desorption and reusability of the synthesized adsorbent was evaluated. The stability of the impregnated AgNPs on the biomass support, as well as the influence of interfering heavy metals in the simulated wastewater on the adsorption of BB, were also considered.

## Materials and methods

### Chemicals

*Solanum tuberosum* was purchased from Checkers, Mimosa Mall, Nelson Mandela Drive, Bloemfontein, South Africa. Bromophenol blue (C_19_H_10_Br_4_O_5_S), silver nitrate (AgNO_3_), sodium hydroxide (NaOH; 97%), cadmium chloride monohydrate (CdCl_2_.H_2_O; 98%), nickel(II) acetate tetrahydrate (Ni(CH_3_COO)_2_.4H_2_O; 98%), hydrochloric acid (HCl; 37%) and nitric acid (HNO_3_; 70%) were obtained from Sigma-Aldrich. Tri-sodium citrate dihydrate (C_6_H_5_Na_3_O_7_.2H_2_O), lead nitrate (Pb(NO_3_)_2_), zinc sulphate (ZnSO_4_), were obtained from Fluka. Without any purification, these chemicals were used as purchased.

### Biomass-nanohybrid preparation

The peels of the purchased *Solanum tuberosum* were removed manually by a kitchen knife, followed by the removal of surface impurities by washing with tap water. The peels were cut into smaller sizes to increase the surface area for drying, then sun dried for 48 h. The sundried peels were dried further in an oven (Labcon model) at 80 °C for 24 h, then pulverized into fine powder with a laboratory pestle and mortar. To ensure the elimination of water-soluble components that could contaminate the water when used for trapping of BB, the sample was further treated with acid. This was done by contacting the biomass powder with 0.1 M HNO_3_ in a beaker with constant stirring for 1 h. The mixture was then washed with excess distilled water until the pH was neutral. Thereafter, the mixture was again dried in an oven at 70 °C for 24 h, and then pulverized with the mortar and pestle, before sieving through 100-µm mesh. The powdered *Solanum tuberosum* peel, designated as biosorbent STpe, was covered in a sample bottle and kept in a desiccator until use.

Incipient wet-impregnation method was used to prepare the biomass-AgNP hybrid, employing both biogenic and chemically assisted reduction of the silver. This was performed by preparing 200 mL of a 25 mM AgNO_3_ solution, which was stirred in a beaker at 30 °C with a magnetic stirrer on a hot plate for 30 min. 8.0 g of pulverized STpe (without nitric acid pretreatment) was then added to the solution with continuous stirring for 2 h. This enabled the reduction of the silver ions by the reducing agent present in the biomass plant material. Followed by the addition of 3 mL of 1% trisodium citrate to further aid the reduction of AgNO_3_ to silver nanoparticles (AgNPs) impregnated onto STpe. The mixture was stirred continuously for 8 h, after which it was allowed to settle for 30 min, followed by centrifugation at 8000 rpm for 1 h. The obtained hybrid was washed with distilled water until neutral pH and the centrifugation was repeated. The composite was then dried in an oven at 70 °C for 24 h. Thereafter it was pulverized thoroughly into fine powder using the mortar and pestle and sieved through a 100-µm screen, designated as biosorbent STpe-AgNP and stored in an air-tight container kept in a desiccator until use. Based on the yield, the ratio of AgNP to STpe in the as-prepared hybrid was 1:9.

### Characterization of the prepared adsorbents

The prepared adsorbents, pristine STpe and STpe-AgNP hybrid, were analyzed to examine the surface properties. The Fourier transform infrared (FTIR) spectrum was obtained using an FTIR spectrometer (Brucker Model). The pH point of zero charge (pHpzc) was evaluated by the pH drift method^33^. X-ray diffraction (XRD) of the structure was determined using an X-ray diffractometer, with Cu radiation of 1.54 Å on a flat plate sample holder. Diffraction patterns were in the 2-theta range of 10–80°, with step size of 0.1° and 2 s counting time per step. The morphology of the two biosorbents was examined by field emission scanning electron microscopy (SEM; Jeol JSM-7800F model), coupled with energy dispersive X-ray spectroscopy (EDX; Oxford X-max 80 mm^2^), to obtain the elemental composition. Thermal stability was evaluated with the thermo-gravimetric analyzer (TGA; SDTA851^e^ Mettler Toledo Model), with 10 °C/min heating rate and 200 ml/min nitrogen flow rate. The specific surface area (S_BET_) and pore properties were evaluated by the surface area and porosity analyzer (Micromeritics ASAP 2020 model) via the Brunauer–Emmett–Teller (BET) isotherm and the results were refined by MicroActive VI.01 software.

### Decontamination of BB solution

A 100 g/L bromophenol blue (BB) stock solution was prepared from C_19_H_10_Br_4_O_5_S in a 250 mL volumetric flask. Solutions of lower BB concentrations of 10–50 mg/L were prepared by dilution. Batch adsorption technique was utilized to evaluate the two adsorbents’ removal potentials for BB, by studying the influence of varying pH (2.0–9.0), BB concentration (10–50 mg/L), biosorbent material dosage (0.02–0.1 g), temperature (300–323 K) and sonication time (5–120 min) agitation by sound energy, on the adsorption percentage. Firstly, the pH was adjusted using a 0.1 M solution of either HCl or NaOH as required. The varying pH experiment was conducted by adding 0.06 g of the prepared biosorbent materials to 10 mL of the simulated BB wastewater, which was placed in an ultrasonic 2.5 L water filled bath and sonicated at 300 K for 120 min. At the end of the given sonication time, the mixture was centrifuged at 8000 rpm for 25 min, and the filtrate was analyzed for BB concentration, using the UV spectrophotometer (Shimadzu UV-1800 model) at maximum wavelength of 590 nm. Other operating factors were kept constant, while changing the respective studied factor of interest. The optimal trapping of BB was conducted at optimum values of pH 4.0, adsorbent dose 0.06 g, concentration 50 mg/L and sonication time of 120 min. The uptake capacity q_e_ (mg/g) and percentage adsorption were calculated from the mass balance and removal percentage equations respectively, as described previously^58^.

### Regeneration and reusability of STpe and STpe-AgNP

Desorption and subsequent reusability of the adsorbent was analyzed by first contacting 0.1 g of the adsorbent material with 10 mL of the 50 mg/L BB solution at optimal pH 4.0, temperature of 300 K and sonication time 120 min. The solution was centrifuged as described previously and the residual concentration of BB was evaluated with the UV spectrophotometer, to deduce the percentage BB adsorbed (q_e_). The BB-loaded adsorbent was then oven dried at 80 °C for 40 min, after which it was contacted with 10 mL of 0.5 M NaOH desorbing solution and sonicated for 30 min at 300 K. The amount of BB in the desorbed alkaline supernatant (C_D_) was then analyzed after centrifugation. The percentage of BB desorbed was then calculated from the equation^59^:

1 $$ \% \, Desorption = 100\left[ {C_{D} V_{D} } \right]/q_{e} m $$

where q_e_ (mg/g) denotes the abstraction capacity of BB by the adsorbents after the initial adsorption conducted, m (g) represents the mass of the biomass material, C_D_ (mg/L) is the amount of BB in the alkaline desorbed eluent solution after elution and V_D_ (L) is the eluent volume utilized for removing the adsorbed BB dye from the loaded biomass material surface. The regenerated adsorbent was then reused for another adsorption after drying in the oven for 30 min at 80 °C. The second adsorption was performed, again utilizing 50 mg/L of BB concentration, sonication time 120 min, pH 4.0 and temperature of 300 K. The biosorbents’ second uptake capacity for BB was again evaluated from Eq. (1). Three adsorption–desorption cycles were conducted to evaluate the reusability of the as-prepared materials for BB adsorption.

### Isotherm modeling

The affinity between BB and the prepared adsorbents was analyzed by the application of the Temkin, Freundlich, Langmuir and Scatchard isotherms^58,60^. The Freundlich isotherm, which relates to a heterogeneous multilayer abstraction, is given as:

2 $$ log\;q_{e} = log\;K_{F} + \left[ {1/n} \right]log\;C_{e} $$

The Langmuir model, which represents a homogenous monolayer adsorption, is expressed as:

3 $$ C_{e} /q_{e} = 1/q_{L} K_{L} + C_{e} /q_{L} $$

The Scatchard model provides verification of either the heterogeneous or the homogenous nature of the material surface and is given as:

4 $$ q_{e} /C_{e} = q_{s} b{-}q_{e} b $$

The Temkin isotherm assumes that the surface coverage of adsorbent is dependent on the removal.

energy and is given as:

5 $$ q_{e} = B\;lnA + B\;lnC_{e} $$

where *n* and *K*_*F*_ (L/g) denote the Freundlich adsorption intensity and capacity respectively, *C*_*e*_ (mg/L) is the BB concentration at equilibrium, *K*_*L*_ (L/mg) is the Langmuir constant and *q*_*L*_ (mg/g) corresponds to the maximum monolayer uptake. The constants *b* and *q*_*s*_ (mg/g) are the Scatchard adsorption parameters, while *A* (L/mg) and *B* correspond to the Temkin’s adsorption binding energy and heat energy respectively.

### Kinetic modeling

The kinetics of BB removal onto pure STpe and the STpe-AgNP hybrid were analyzed by the pseudo-first order (PFO), pseudo-second order (PSO), liquid film diffusion (LFD) and intraparticle diffusion (ID) kinetic equations^61^.

The PFO equation is expressed as:

6 $$ \log \left( {q_{e} - q_{t} } \right) = \log q_{e} - \frac{{k_{1} }}{2.303}t $$

The PSO kinetic equation is given as:

7 $$ \frac{t}{{q_{t} }} = \frac{1}{{k_{2} q_{e}^{2} }} + \frac{t}{{q_{e} }}e $$

The LFD equation used to analyze the mechanism of diffusion is expressed as:

8 $$ ln\left( {1{-}F} \right) = Y{-}k_{FD} t $$

The ID also applied to provide information on adsorption diffusion mechanism is expressed as:

9 $$ q_{t} = k_{d} t^{1/2} + C $$

where *t* (min) is the sonication time of sound energy agitation, *q*_*t*_ (mg/g) is the uptake capacity at a particular time, *F* = *q*_*t*_*/q*_*e*_ represents the fractional equilibrium attainment. The constants *k*_*1*_ (min^−1^), *k*_*2*_ (g/mg min), *k*_*FD*_ (mg/g min) and *k*_*d*_ (mg/g min^1/2^) represent the PFO, PSO, LFD and ID rate constants respectively. *Y* and *C* are the intercepts of the LFD and ID equations respectively, which show the deviation of the plots from the origin.

## Thermodynamic analysis

Adsorption thermodynamics analysis was performed to evaluate the enthalpy change (ΔH°), entropy change (ΔS°) and Gibbs free energy change (ΔG°) by the application of the given equations^62^:

10 $$ \Delta G^{o} = - RT \, lnK_{c} $$

11 $$ ln \, K_{c} = - \left( {\Delta H^{ \circ } / \, RT} \right) + \left( {\Delta S^{ \circ } /R} \right) $$

where symbols *R* (J/mol K), *T* (K) and *K*_*c*_ correspond to the ideal gas constant, absolute temperature and distribution coefficient respectively.

## Statistical evaluation of isotherm and kinetic models

The best-fit isotherm or kinetic model was evaluated by the nonlinear chi-square (χ^2^) and regression coefficient (R^2^). The R^2^ was determined by the statistical function of Microsoft Excel, 2010. The best model fit is for the R^2^ value closest to one and for the lowest χ^2^ value. The χ^2^ value was calculated from the equation:

12 $$ \chi^{2} = \sum \left[ {\left( {q_{e(cal)} {-}q_{e(exp)} } \right)^{2} /q_{e(exp)} } \right] $$

where *q*_*e(exp)*_ and *q*_*e(cal)*_ in mg/g, correspond to the experimental and calculated adsorption capacities respectively^63^. The experiments were performed in triplicate and the mean values were computed for quality assurance. The errors bars in the figures represent the standard deviation from the mean.

## Results and discussion

### FTIR and XRD analysis

**Figure 1:**
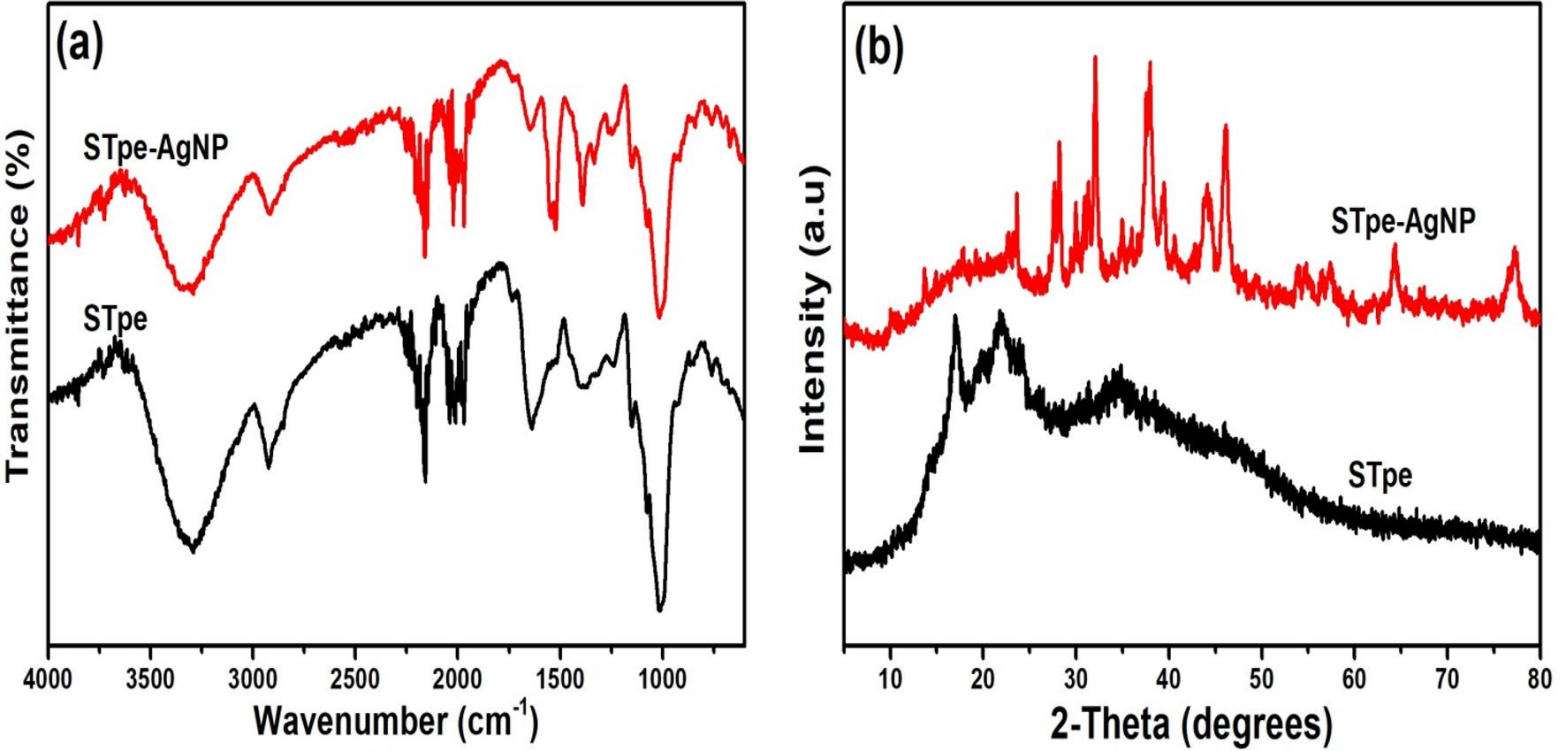
(**a**) Fourier transform infrared spectra (FTIR) investigating the presence of surface functional groups and (**b**) X-ray diffraction analysis (XRD) of both biosorbents, with only the STpe-AgNP hybrid showing crystalline peaks of the impregnated Ag nanoparticles.

The surface functional groups on both STpe and STpe-AgNP were evaluated as depicted in the Fourier transform infrared (FTIR) spectra (Fig. 1a). Changes in FTIR absorption bands of the two adsorbents after surface alterations, provided insight into the surface functional groups utilized in the synthetic process of silver nanoparticle impregnation. The FTIR spectrum of pristine STpe showed a broad OH band at 3291 cm^−1^^33^. The C–H stretching of alkenes and alkanes was observed by IR absorption bands at 2923 cm^−1^, while the C≡C group corresponded to IR absorption bands at 2199 and 2049 cm^−1^^29,33^. Absorption at 1638 cm^−1^ was attributed to carbonyl C=O stretching, with the C=C band of alkenes observed at 1374 cm^−1^^33,53^. The absorption bands at 1154 cm^−1^ and 1003 cm^−1^ were due to the C–O stretching vibration^33,59^. After impregnation of AgNP onto the STpe adsorbent forming the hybrid (STpe-AgNP), slight shifts in the IR absorption bands from 2923 to 2917 cm^−1^, 2199 to 2193 cm^−1^, 2049 to 2021 cm^−1^, 1638 to 1623 cm^−1^, 1374 to 1389 cm^−1^ and 1003 to 998 cm^−1^ frequencies, suggested interactions of the corresponding functional groups on STpe with the attached AgNPs. The appearance of the N–O band at 1530 cm^−1^ could be due to the reaction between the nitrate of AgNO_3_ and the biosorbent. Many interactions were suspected in the C=O region as the broad IR absorption band became sharper after nanoparticle incorporation. However, the fact that all functional groups were retained, including the OH at 3291 cm^−1^, showed that AgNP impregnation did not alter the surface functional groups of the pristine STpe, which are important for efficient sequestration of BB molecules from solution.

The X-ray diffraction analysis (XRD) of both adsorbents is presented in Fig. 1b. The diffraction pattern depicted by STpe clearly conforms to the amorphous nature of lignocellulosic materials^33,64^. However, crystalline peaks were displayed in the diffraction patterns exhibited by STpe-AgNP attributed to the impregnation of the silver nanoparticles (AgNPs) onto the biosorbent. The diffraction pattern of the STpe-AgNP hybrid showed four distinct diffraction peaks of silver at 2θ values of 38.36°, 44.31°, 64.65° and 77.23°, corresponding to the cubic lattice silver structural planes of (111), (200), (220) and (311) respectively, according to JCPD89-3722. Other diffraction peaks ascribed to AgCl were also observed, which formed because of the reaction between Ag ions from AgNO_3_ with the chloride content of the STpe biomass. The occurrence of AgCl diffraction peaks along with silver diffraction was also observed by some other researchers^65,66^. Importantly, the transformation of the STpe diffraction pattern after AgNP impregnation clearly indicates successful impregnation of the nanoparticles onto the pristine biosorbent. The average crystalline size of the AgNP on the hybrid was calculated from the Debye–Scherrer equation, expressed as^34^:

13 $$ D = k\lambda /\beta cos\theta $$

where *D* represents the average crystalline size of AgNP; λ is the wavelength of X-ray used; k is the geometric factor equivalent to 0.9; while *β* represents the full width of the half-maximum diffraction peak (FWHM). The value of D was calculated as 21.94 nm, which indicated smaller crystalline size AgNP nanoparticles on the hybrid, when compared to the size of 25 nm reported by Dawodu et al.^34^, but quite larger than the sizes of 15.77, 16.98 and 19.94 nm reported in another study by Ukkund et al.^67^. These small crystalline particles could probably be attributed to efficient stabilization of the AgNPs by the biogenic components of the biomass STpe, preventing aggregation of the nanoparticles.

### SEM, EDX and particle size distribution

**Figure 2:**
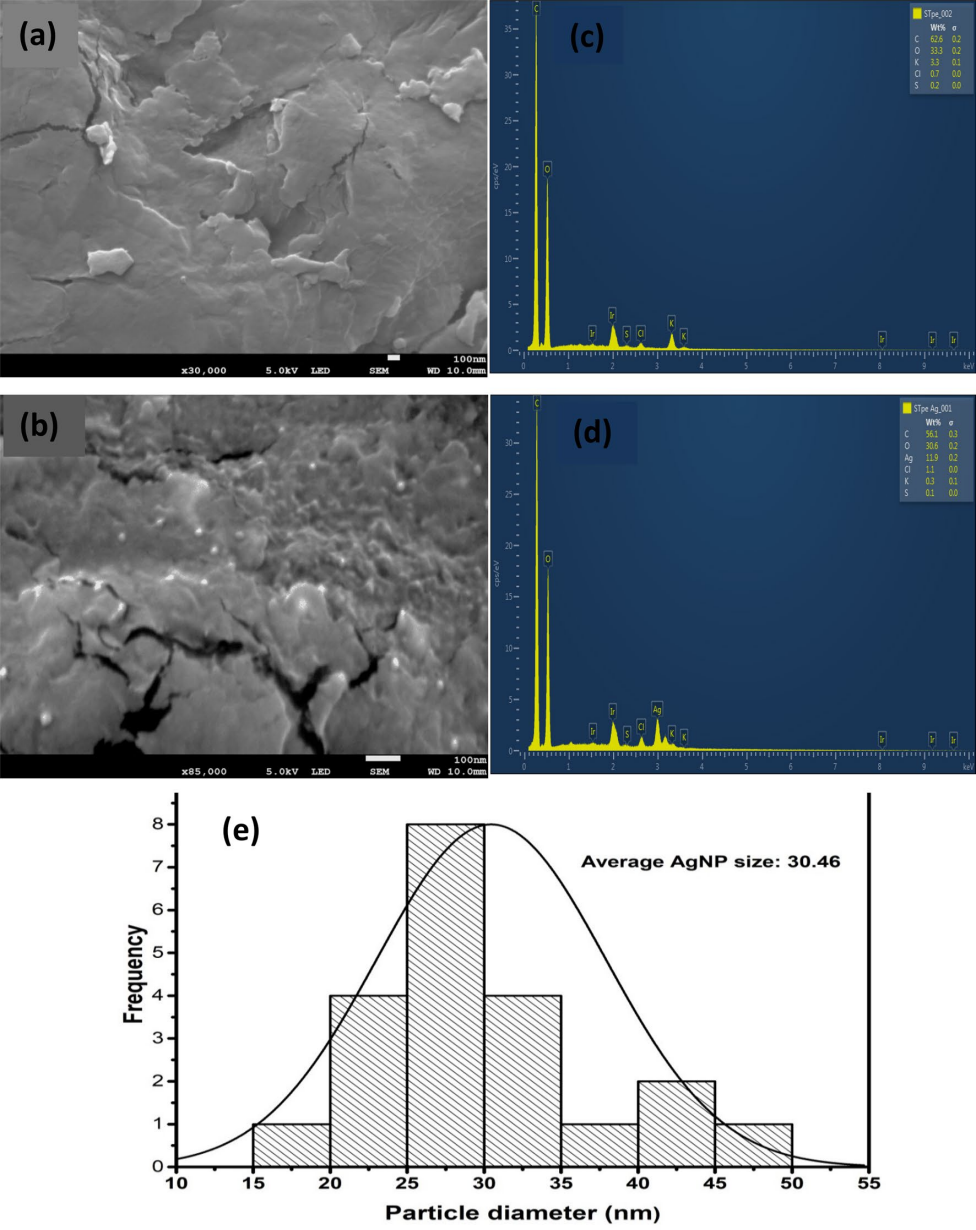
Scanning electron microscopy (SEM) of adsorbents (**a**) STpe and (**b**) the STpe-AgNP hybrid, showing increased roughness in surface morphology in the latter; and Energy dispersive X-ray spectroscopy (EDX) of (**c**) STpe and (**d**) the STpe-AgNp hybrid, showing their elemental compositions; and (**e**) particle size distribution of the AgNP nanoparticles on the hybrid adsorbent.

The surface morphology of our prepared adsorbents was examined by field emission scanning electron microscopy (SEM), as shown in Fig. 2a,b. The SEM image of pure STpe displayed a slightly rough surface morphology without particle distribution^33^. However, after impregnation with silver, STpe-AgNP showed increased surface roughness, due to the AgNPs distributed on the hybrid adsorbent surface. The AgNPs looked somewhat spherical in shape with a white appearance on the surface of STpe, similar to that obtained for AgNP-activated carbon hybrid^53^. The absence of white spherical particles in the image of the pristine STpe clearly indicates successful impregnation of AgNPs in the hybrid material. This was further supported by the changes in the energy dispersive X-ray (EDX) elemental compositions of the adsorbents, before and after AgNP impregnation. The EDX analysis of pristine STpe (Fig. 2c) showed compositions of C (62.2%), O (33.3%), K (3.3%), Cl (0.7%) and S (0.2%)^33^. The EDX analysis of STpe-AgNP (Fig. 2d) was C (56.1%), O (30.6%), Ag (11.9%), Cl (1.1%), K (0.3%) and S (0.1%), which showed an appreciable amount of silver. In addition, the presence of chloride in the original STpe corroborated our XRD results of the existence of an AgCl diffraction pattern observed in the STpe-AgNP hybrid, which was attributed to the reaction of silver and the chlorine present in the potato peel biomass. In addition, the particle size distribution of the AgNPs from the morphology of STpe-AgNP (Fig. 2e) showed particles in the range of 15–50 nm, with an average size of 30.46 nm. This was in close agreement with the deduction from XRD analysis, where an average crystalline size of 21.94 nm was obtained from the Debye–Scherrer Eq. (13), suggesting small size AgNPs in our prepared hybrid adsorbent.

### BET and TGA analysis

**Figure 3:**
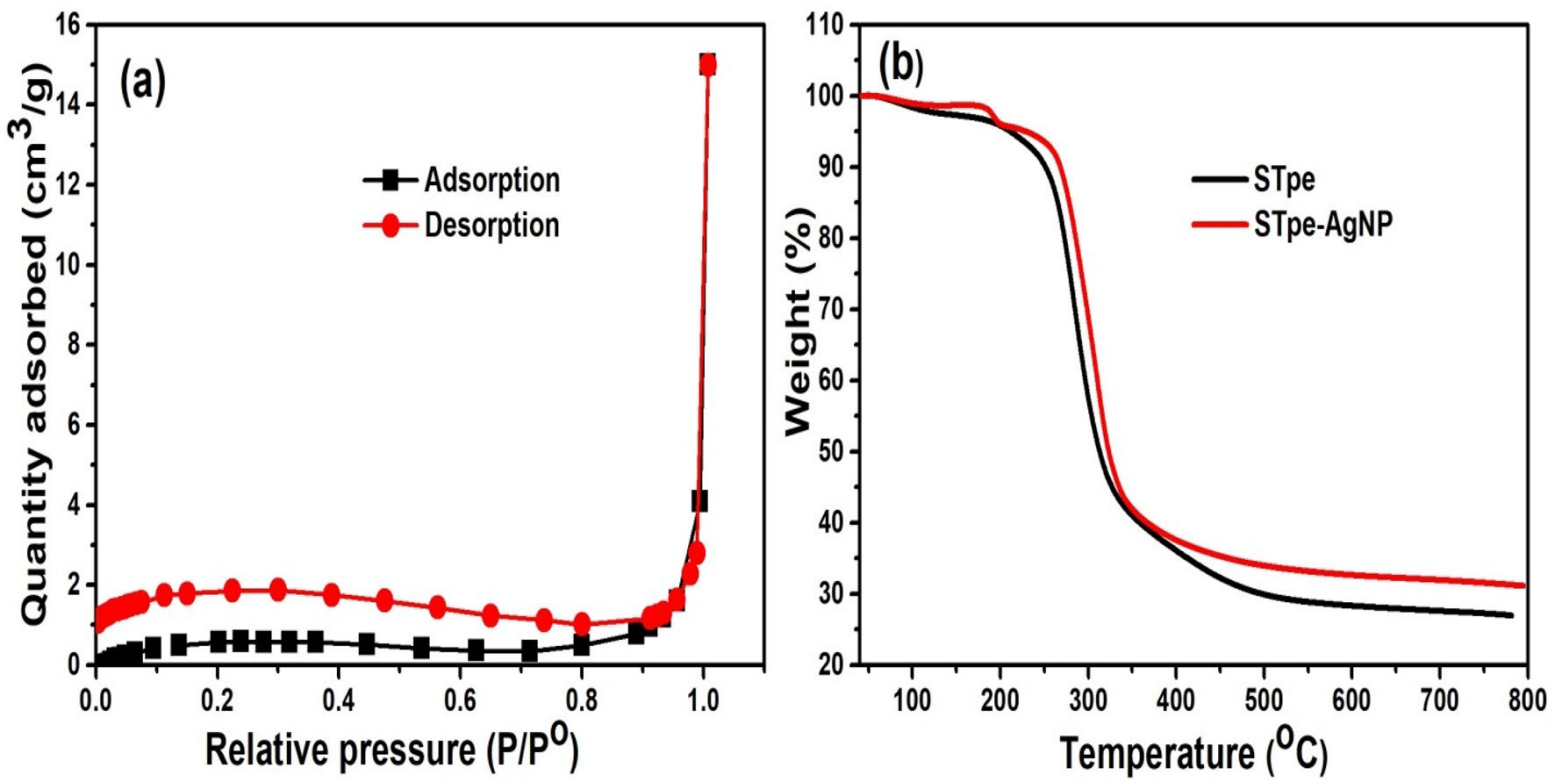
(**a**) Nitrogen adsorption–desorption isotherm of the STpe-AgNP hybrid, investigating the S_BET_ specific surface area and pore volume; and (**b**) Thermo-gravimetric analysis (TGA) of both adsorbents, showing their thermal stability.

Additional information on the surface properties of materials were also obtained from the Brunauer–Emmett–Teller (BET) specific surface area and porosity analysis. The N_2_-adsorption–desorption isotherm analysis of STpe revealed a BET surface area (S_BET_) of 8.38 m^2^/g and pore volume of 0.0174 cm^3^/g^33^. The low surface area observed is often a characteristic of most agro-materials. However, after the impregnation of silver nanoparticles onto STpe biomass, the N_2_-adsorption–desorption analysis of STpe-AgNP (Fig. 3a) showed a very slight increase in the S_BET_ surface area to 9.13 m^2^/g. This increase is desirable because it is generally believed that higher surface area implies higher removal of pollutants from solution, but that is not always the case. The fact that only a negligible change in surface area was observed, could also mean only a corresponding slight enhancement in pollutant uptake onto the material^25^. Furthermore, a STpe-AgNP pore volume of 0.0028 cm^3^/g was obtained, indicating a decrease of pore volume after nanoparticle modification. This decrease could be attributed to the presence of AgNPs on the pores of STpe. Notwithstanding, the nanoparticles provide additional surface characteristics to the biosorbents, which are known to influence their surface interaction with the adsorbate. Similar findings have been documented in the removal of mercury(II) contaminants from wastewater, by a silver/quartz nano-composite adsorbent^50^.

The thermal stability of adsorbent materials is important as it provides information on their behaviour under various temperature conditions. A thorough search revealed that most studies on silver nanoparticle impregnation on adsorbents did not consider the thermal stability of the materials. We thus investigated the stability of both STpe and STpe-AgNP with temperature, by application of the thermo-gravimetric analysis (TGA) as shown in Fig. 3b. From the thermogram, a slight weight loss of both adsorbents was recorded from 40 to 250 °C, attributed to the removal of moisture and volatile components from the adsorbents^29^. This was followed by significant weight loss from 250 to 450 °C, mainly due to decomposition of organic matter^68^. Most importantly, it was evident from the TGA that the impregnation of AgNPs onto STpe slightly increased the thermal stability of the material. This was observed from the lower weight loss of the STpe-AgNP hybrid at all temperatures, compared to pure STpe. Also at 750 °C, the hybrid adsorbent retained more than 33% of the initial weight, while STpe retained about 26%. This indicated that the hybrid material would be more stable for pollutant removal involving high temperature conditions, compared to the pristine adsorbent.

### Stability of impregnated AgNP on the hybrid adsorbent

**Figure 4:**
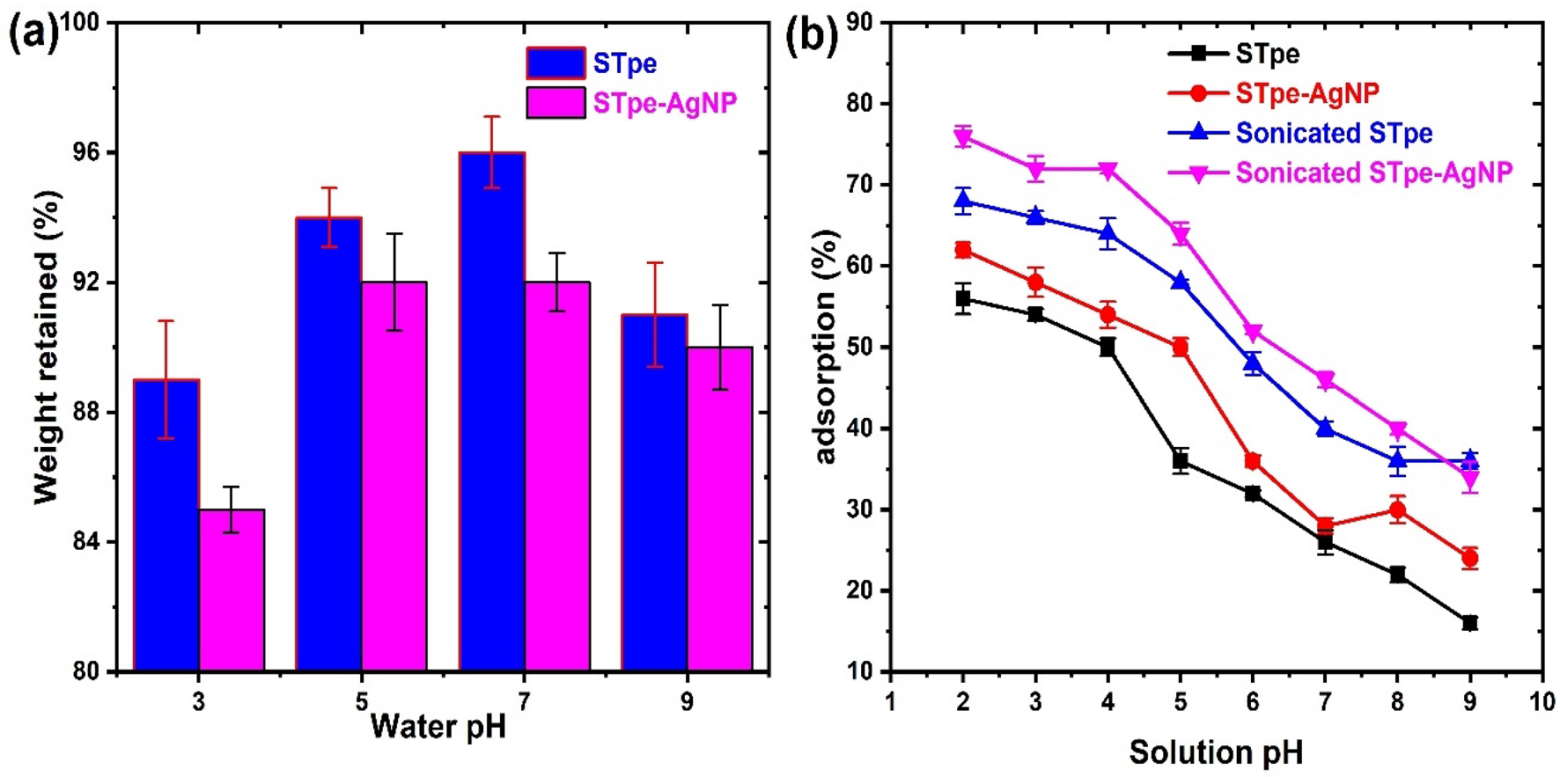
(**a**) Stability of the silver nanoparticles on the STpe biomass support in water at various pH values, and (**b**) the effect of solution pH of the wastewater and agitation by ultra-sonication on the adsorption of bromophenol blue (at pH 4.0, adsorbent dosage 0.06 g, temperature 300 K, dye concentration 50 mg/L, sonication time 120 min, adsorbent contact time 180 min).

The stability of the nanoparticles on the STpe biomass support was examined. This was performed due to the possibility of the AgNPs to be removed from the hybrid into solution during the adsorption process. There is a high tendency for such a situation to occur if the attached nanoparticles are only held by weak forces onto the biomass support. We evaluated the stability by contacting 0.06 g of STpe-AgNP with 10 mL of distilled water in 100 mL conical flasks each adjusted to pH values of 3.0, 5.0, 7.0 and 9.0. The mixture was sonicated at room temperature in an ultrasonic bath with ultrasound frequency of 30 kHz for 80 min. This was followed by filtration using a Whatman No.4 filter paper (Diameter 125 mm). The dry mass of STpe-AgNP on the filter paper was determined and the percentage weight retained was calculated. The AgNPs removed from the hybrid into the solution would likely pass through the pores of the filter paper, taking into consideration the average particle size of 30.46 nm obtained. On the other hand, the AgNP loaded onto the STpe biomass support would be retained on the paper. We also performed the same experiment using STpe alone as a control, since there is a possibility for loss of certain components from STpe into the solution, which would wrongly be attributed to the AgNP removal. The stability result of the adsorbents is presented in Fig. 4a. We observed that pure STpe retained up to 96 and 93% initial weight at pH 7.0 and 5.0 respectively, with 89% at more acidic (pH 3.0) and 91% at more alkaline (pH 9.0) conditions. The removal of more STpe components could be associated with acid and alkaline activation of the biosorbent^29^, with higher stability at neutral pH. For STpe-AgNP, which is our adsorbent of interest, weight retained of 85, 92, 92 and 90% was obtained at pH 3.0, 5.0, 7.0 and 9.0 respectively. Little weight loss of our hybrid adsorbent, with values very close to the pristine STpe was thus observed, with the most stable at neutral pH. This result suggests that the impregnated AgNPs are stable on the hybrid, which is desirable during the adsorption of dye molecules.

### Effect of solution pH and sonication on BB removal

The solution pH of the wastewater affects the adsorbent and adsorbate species in any adsorption study. This is due to the protonation or deprotonation of the functional groups, both of the adsorbate and adsorbent, thereby altering the surface charge or speciation at various pH values^8^. Electrostatic interactions can then be created between the charged species during the adsorption process. Solution pH is thus an important parameter affecting adsorption and needs to be considered. The relationship between the equilibrium percentage removal of BB by the adsorbents and the pH of solution is illustrated in Fig. 4b. A steady decrease in BB adsorption on both STpe and STpe-AgNP was obtained as the pH increased from 2.0 to 9.0. Anionic BB molecules in solution at acidic pH values were not likely to encounter repulsions from the positively charged acidic protons, for binding onto the adsorbent surface. The pKa of BB was 3.84, hence the BB exists mainly as negatively charged species up until pH 3.84, after which the BB existed as neutral molecules and at high pH as slightly positive charged species. In addition, the pHpzc of STpe and STpe-AgNP was found to be 6.2 and 6.8 respectively. This implies that at pH values lower than 6.2 and 6.8 for the respective adsorbents, the adsorbent surfaces are positively charged, while they are negatively charged at values greater than the pHpzc. Therefore, simply put, the highest adsorption observed at lower pH values was due to electrostatic attraction between the negatively charged BB species and the positively charged adsorbent surfaces^25^. A similar result was also obtained in the adsorption of BB onto modified *Hermetia illucens* larvae biosorbent^11^. Although solution pH of 2.0 gave the highest adsorption, yet in this study a constant pH = 4.0 was chosen for the subsequent adsorption experiments, because the differences in adsorption from pH = 2.0 to 4.0 varied only very slightly, as well as the significant fact that pH = 4.0 lies within the real practicable pH range of contaminated wastewaters. Various researchers recorded different trends of pH influence on the adsorption of BB onto different adsorbents^6,9,69^, which suggest that the nature of the adsorbent itself plays the major role in BB adsorption.

In addition, this study analyzed the influence of ultra-sonication by sound wave agitation on the removal of BB by the adsorbents, as shown in Fig. 4b. Significant improvement in adsorption with sonication was recorded, compared to the adsorption without sonication. This implied that ultra-sonication of the solution during the adsorption process favoured BB removal onto STpe and STpe-AgNP. The ultrasonic mechanical waves form unique suspensions in solution, causing improved surface contact between the dye molecules and the biomass materials, enhancing removal by the biosorbents^70^. We thus performed all subsequent experiments by applying ultrasonic waves to enhance the removal of BB from solution. Another interesting observation was the fact that the STpe-AgNP hybrid exhibited higher removal of BB dye than the pristine STpe, within the studied pH range. This indicates that the impregnation of AgNPs onto the STpe biomass created more efficient interaction of the hybrid material with BB, compared to the pristine biosorbent.

### Effect of some operating variables on adsorption

**Figure 5:**
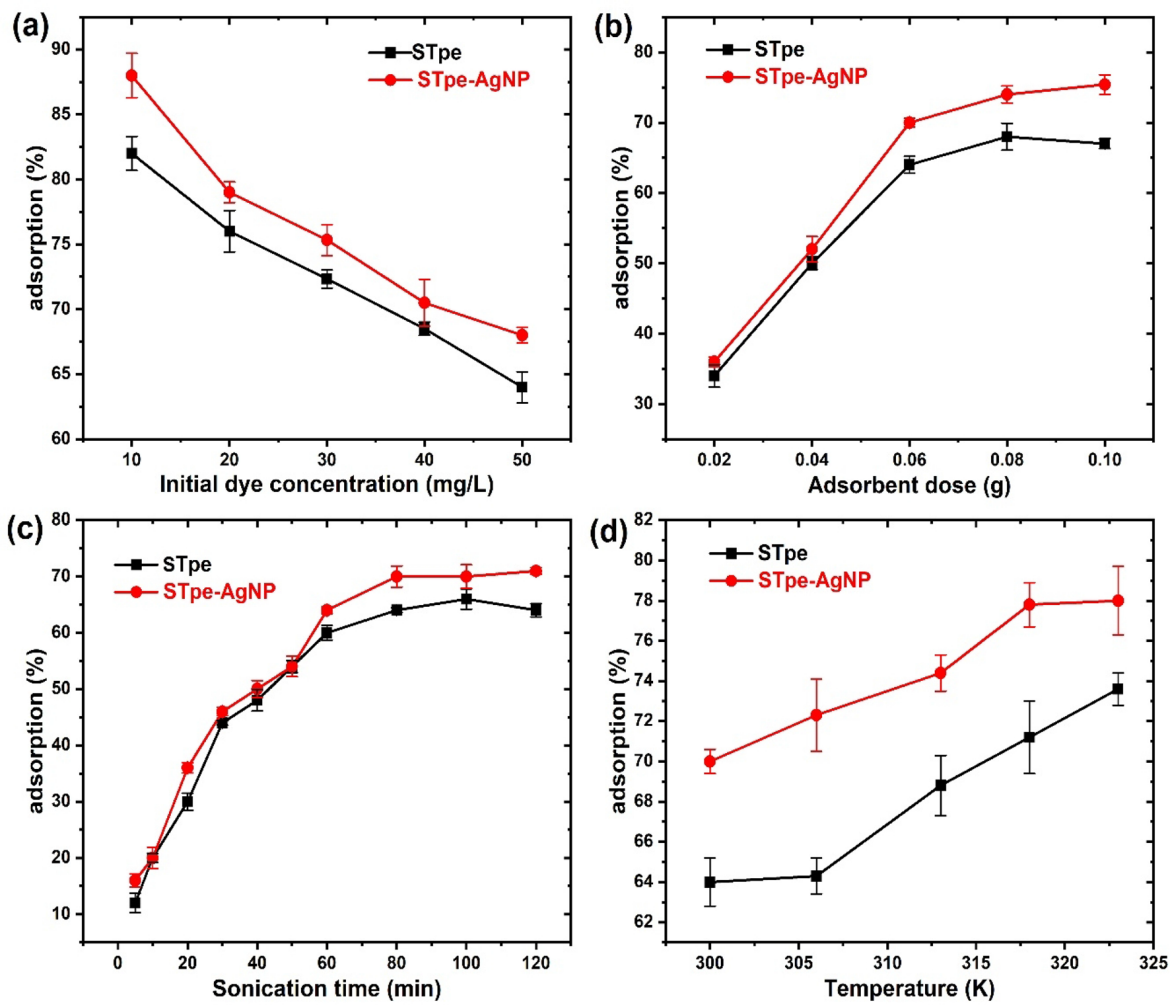
Influence of (**a**) initial bromophenol blue concentration on adsorption (with constant pH 4.0, dosage 0.06 g, sonication time 120 min, Temp. 300 K); (**b**) of biomass adsorbent dosage on adsorption pH 4.0, dye conc. 50 mg/L, sonication time 120 min, Temp. 300 K; (**c**) of sonication time on adsorption (pH 4.0, dosage 0.06 g, dye conc. 50 mg/L, Temp. 300 K; and (**d**) of solution temperature on adsorption (pH 4.0, dosage 0.06 g, dye conc. 50 mg/L, sonication time 120 min kept constant).

Apart from solution pH, several other factors are known to also influence the adsorption process of pollutants from wastewater. These factors are mainly the adsorbate (BB dye) concentration, dosage of the biomass adsorbent, adsorption time and temperature of the solution. We thus considered the influence of these factors on the sequestration of BB from solution. The dependence of initial BB concentration and its adsorption on STpe and STpe-AgNP is shown in Fig. 5a. A steady decrease in the abstraction percentage of BB onto both adsorbents with increasing initial concentration of the BB dye, was displayed. There is a fixed number of active sites on the adsorbent surface, since the biomass adsorbent dosage used remains constant at 0.06 g throughout for the varying BB concentration of 10–50 mg/L. Thus, the ratio of BB/adsorbent is low at lower initial concentration of BB. There is a progressive increase in the ratio of BB/adsorbent as the initial dye concentration increases; this correspondingly increases the competition of BB for the available adsorption sites on the biomass material, leading to lower removal percentage. Also, this trend could be attributed to increasing hindrance from mass transfer resistance as a result of increasing solution viscosity as BB concentration increases^71^. STpe-AgNP showed slightly higher adsorption of BB than pure STpe at the range of BB concentration studied, which indicates that the impregnation of AgNPs onto STpe slightly increased the adsorption potential.

The adsorption of BB onto both adsorbents and its dependence on biomass material dosage, at a fixed BB concentration of 50 mg/L, is illustrated in Fig. 5b. Significant increase in percentage adsorption of BB dye onto both adsorbents, with increasing biomass dosage from 0.02 to 0.06 g, was achieved. Thereafter only slight changes in adsorption were observed from 0.06 to 0.10 g biomass. We therefore chose 0.06 g as the constant biomass dosage, when evaluating the effect of other operational parameters on BB trapping. The reason for the initial sharp increase in BB adsorption in Fig. 5b, is due to the increasing number of available adsorption sites for efficient interaction, as the biomass dosage increases^6^. The latter only slight changes or constant trend could be attributed to aggregation of active adsorption sites at higher dosage of biomass adsorbent. A similar trend was observed by other scientists^9,69^. The hybrid material also recorded slightly higher adsorption than pristine STpe, with a more significant adsorption difference above 0.06 g biomass dosage, indicating enhanced removal of BB with AgNP impregnation of the potato peel.

An efficient adsorbent should not only be associated with high uptake capacity for the adsorbate, but in addition should also have a fast rate of adsorption. This is because a faster adsorption rate saves a lot of time, which is important for economic operation of water treatment plants. In this regard, the variation of BB adsorption with sonication time was investigated, as shown in Fig. 5c. A fast adsorption of BB onto both STpe and STpe-AgNP adsorbents was achieved within the first 30 min. After that, a slower uptake was observed up to 80 min, after which the removal rate became constant at equilibrium. The large amount of available adsorption sites and the high initial BB concentration account for the rapid initial uptake recorded. As adsorption progresses, the adsorption sites get used up and the BB concentration in solution decreases, leading to the slower removal. The constant or insignificant removal at the final equilibrium stages is due to saturation of adsorbent sites^72^. Similar trends have been observed by other researchers^73,74^. Both STpe and STpe-AgNP showed very similar adsorption of BB, especially from 5 to 60 min, after which the latter increased slightly more than the pristine STpe. Equilibrium was achieved around 80 min on both adsorbents, although a constant sonication time of 120 min was used throughout our experiments to ensure equilibrium was attained. This result indicated that AgNP impregnation on the STpe biomass support did not increase the rate of removal of BB from solution.

The temperature of the wastewater is also an important factor influencing the uptake of most pollutants from solution. In this context, the dependence of BB abstraction onto STpe and STpe-AgNP as a function of temperature was determined, as shown in Fig. 5d. Increasing solution temperature from 300 to 323 K, increased the abstraction of BB from 64 to 73% onto STpe and from 70 to 78% onto STpe-AgNP respectively. This clearly indicated that higher temperatures favor the adsorption of BB onto both adsorbents and the process is endothermic. This adsorption increase is due to enhanced mobility of BB molecules with higher temperature, for more effective interaction with the active sites on the adsorbent^75^. It is also attributed to the creation of new active sites on the adsorbent surface, due to removal of some surface impurities with temperature increase^76^. The removal of BB from solution by some other researchers also displayed endothermic adsorption^6,9,10,73,77^ similar to this study, while others recorded exothermic adsorption^78–80^ using other biosorbents. The nature of the adsorbent and its active sites is thus a major factor in the effective adsorption of BB as a function of temperature. Furthermore, AgNP impregnation on the STpe biomass support enhanced the adsorptive removal of BB dye at the studied temperature range.

### Equilibrium isotherm evaluation of the process

Isotherm equilibrium analysis provides vital insight into the affinity between the adsorbent material and the pollutant in solution during the treatment process^81^. The isotherm model parameters obtained for the sequestration of BB onto both adsorbents, are presented in Table 1. We deduced that the Langmuir model, which depicts a monolayer removal process of the adsorbate, utilizing homogenous active sites of the adsorbent, resulted in lower R^2^ and higher χ^2^ error values for both adsorbents, compared to the Freundlich model. Thus, the Langmuir model did not provide the best fit for the isotherm equilibrium data, indicating that BB uptake onto the adsorbents is not restricted to a monolayer. It is clear that the Freundlich model produced the best fitting of the isotherm data, as presented by the highest R^2^ and lowest χ^2^ error values for both STpe and STpe-AgNP biosorbents. This indicates that the adsorbents are composed of heterogeneous active sites and their adsorption of BB is a multilayer adsorption^59^. Additionally, a physisorption mechanism of BB waste removal onto the adsorbents is proposed by the better fit of the Freundlich model. This is due to the fact that the Langmuir isotherm is mostly associated with a chemisorption mechanism, although not in all cases. In order to further verify the nature of the materials’ active sites, we also considered the Scatchard model, also called the independent oriented site analysis. The deviation of the Scatchard model from linearity indicates heterogeneous active sites, while high conformation to linearity is indicative of a homogenous adsorbent^58^. The values of R^2^ and χ^2^ obtained for both adsorbents deviate from Scatchard linearity and therefore corroborate the best fit of the Freundlich model, confirming the heterogeneous nature of STpe and STpe-AgNP. Although pristine STpe demonstrated a good fit with R^2^ of 0.9494, yet the high error χ^2^ of 3.64 suggests deviation from linearity in this oriented site analysis. The STpe-AgNP hybrid on the other hand showed even greater deviation, with R^2^ of 0.8143 and χ^2^ of 5.21, which means that AgNP impregnation onto STpe increased the heterogeneous nature, by providing additional AgNP sites. Lastly, the Temkin model, which assumes that the materials’ surface coverage is a function of the adsorption free energy, exhibited lower R^2^ and higher χ^2^ error values than both the Langmuir and Freundlich isotherms and was therefore not analyzed further.

**Table 1:** Isotherm model parameters for the adsorption of bromophenol blue onto pure STpe and STpe-AgNP hybrid.

Isotherm	STpe	STpe-AgNP
Langmuir		
K_L_ (L/mg)	0.101	0.118
q_L_ (mg/g)	8.16	9.6
R^2^	0.9886	0.9522
χ^2^	0.82	0.97
Freundlich		
N	1.66	1.92
K_F_ (L/g)	1.022	1.305
R^2^	0.9968	0.997
χ^2^	0.17	0.23
Scatchard		
b (L/mg)	0.111	0.1915
q_s_ (mg/g)	7.77	6.90
R^2^	0.9494	0.8143
χ^2^	3.64	5.21
Tempkin		
B (mg/g)	1.734	1.577
A (L/g)	1.072	1.718
R^2^	0.9781	0.9458
χ^2^	1.77	2.41

In addition, the consideration of a separation parameter obtained from the Langmuir model provides vital information into the nature of adsorption. The separation parameter (R_L_)^37^ is given as:

14 $$ R_{L} = 1 \, /\left[ {1 \, + \, K_{L} C_{o} } \right] $$

where K_L_ (L/mg) represents the Langmuir separation constant and C_o_ (mg/L) is the initial BB concentration. If the value of R_L_ is equal to one, it depicts linear adsorption, while a R_L_ value of zero is an irreversible adsorption, and R_L_ values greater than one are attributed to unfavourable adsorption^82^. On the other hand, values of R_L_ between 0 and 1 are indicative of a favourable adsorption process^83^. A favourable process was inferred for both biosorbents, from the calculated R_L_ values in the range of 0.165–0.497 for STpe and 0.145–0.459 for STpe-AgNP. Favourable adsorption simply means efficient interaction between BB in the waste solution and the active sites of the adsorbent via a spontaneous process. This implies that both STpe and STpe-AgNP have potential application in the decontamination of BB from polluted water.

The pristine and hybrid adsorbents were further compared with other adsorbents applied previously for the removal of BB from water, as shown in Table 2. The materials were arranged according to decreasing adsorption uptake capacity (q_e_). It can be observed that the prepared materials from this study had lower removal capacity compared to most of the adsorbents from literature and that AgNP impregnation only slightly enhanced the uptake capacity of STpe biomass for BB from solution. However, it is important to note that the biosorbent STpe is more readily available worldwide as biowaste and cheaper than any modified biosorbent or chemically manufactured sorbent.

**Table 2:** Comparison of bromophenol blue adsorption onto pure STpe and STpe-AgNP hybrid, compared with other adsorbents from literature, according to decreasing uptake capacity (q_e_).

Adsorbent	q_e_ (mg/g)	Reference
Modified exuviae of *Hermetia illucens* larvae	564–573	^11^
*Rhizopus stolonifer* biomass	333	^94^
Surfactant modified organo-clays	220.27—400.19	^79^
Supported ionic liquids	217.39	^73^
Activated carbon loaded CuS nanoparticles	106.38	^8^
*Astragalus bisulcatus* tree activated carbon	23.45	^69^
Chitin nanoparticles	22.72	^9^
Reduced graphene oxide	17.94	^77^
Mesoporous hybrid gel	17.67	^7^
Sorel cement nanoparticles	16.39	^6^
Polymer–clay composite	10.78	^10^
*Solanum tuberosum* peel–nanoparticle hybrid (STpe-AgNP)	9.604	This study
*Solanum tuberosum* peel (STpe)	8.157	This study
Magnetic nanoparticle-*Musa acuminata* peel composite	8.12	^25^
*Musa acuminata* peel	6.04	^25^
Polymeric gels	2.99–2.98	^80^
Activated charcoal	0.081	^78^

### Kinetic evaluation of the process

The kinetic rate parameter for adsorbate removal from solution is useful in adsorption system design. We thus evaluated the abstraction kinetics by applying the PFO, PSO, ID and film LFD models. Table 3 presents the kinetic parameters for BB adsorption onto STpe and STpe-AgNP adsorbents. From the R^2^ values obtained, the pseudo-second order (PSO) model exhibited higher values, thus presenting as a seemingly better fit for both adsorbents than the pseudo-first order (PFO) model. Chemisorption mechanism is usually assumed by the best fit of the PSO model to kinetic data. However, the calculated q_e_ uptake capacity (q_e(cal)_) of 6.59 and 6.69 for the PFO onto STpe and STpe-AgNP, respectively, was closer to the experimental q_e_ (q_e(exp)_), than the values presented by the PSO model. In addition, the χ^2^ error values of the PFO model were lower than of PSO for both adsorbents. The χ^2^ helps to minimize the error associated with R^2^, which is encountered due to inherent bias from linearization. The χ^2^ error values usually are applicable when it is becomes difficult to give a clear distinction between model fits. This analysis suggests that the PFO model actually describes the abstraction of BB onto both adsorbents better than the PSO model, and that chemisorption after all is not the likely mechanism of abstraction.

**Table 3:** Kinetic rate model parameters for the trapping of bromophenol blue onto STpe and STpe-AgNP hybrid.

Kinetic model	STpe	STpe-AgNP
Experimental		
q_e(exp)_ (mg/g)	5.5	6.1
Pseudo-first order (PFO)		
k_1_ (min^−1^)	0.043	0.039
q_e(cal)_ (mg/g)	6.59	6.79
R^2^	0.9738	0.9482
χ^2^	0.124	0.365
Pseudo-second order (PSO)		
k_2_ (g/mg min)	3.4 × 10^–3^	4.1 × 10^–3^
q_e(cal)_ (mg/g)	7.88	7.79
R^2^	0.9873	0.9811
χ^2^	0.208	0.377
Intraparticle diffusion (ID)		
k_d_ (mg/g min^−1/2^)	0.682	0.635
C	0.4118	0.0349
R^2^	0.9819	0.9664
χ^2^	0.433	0.374
Liquid Film diffusion (LFD)		
k_fd_	0.043	0.039
Y	0.1819	0.1251
R^2^	0.9738	0.9482
χ^2^	0.817	1.103

The ID and LFD models were used to examine the diffusion mechanism, since such information cannot be obtained from the PFO and PSO models. From the diffusion mechanism analysis, the ID model gave a better fit than the LFD model, as presented by the higher R^2^ and lower χ^2^ values for both STpe and STpe-AgNP. This shows that intraparticle diffusion (ID) is the rate controlling mechanism in the removal of BB onto STpe and STpe-AgNP. However, there is a deviation of the ID plots from the origin, as indicated from the intercept (C). This suggests that ID is not the only diffusion mechanism involved, but there is also some contribution from liquid film diffusion (LFD) to the overall adsorption process^84^. A similar result was documented in the adsorption of Cr(VI) onto magnetite coated biomass^64^. The greater contribution of intraparticle diffusion in the removal of BB dye could be attributed to the ultra-sonication process employed, agitating the particles in solution via high frequency sound waves. Sonication is known to enhance the diffusion of dye onto the biosorbent surface and into the adsorbent active sites, by overcoming mass transfer hindrances^85^. Therefore, sonication provides feasible interaction between BB in solution and the active sites of STpe and STpe-AgNP.

### Thermodynamic evaluation of BB adsorption

Adsorption thermodynamic parameters such as ΔS°, ΔH° and ΔG° were evaluated for the abstraction of BB onto STpe and STpe-AgNP, as presented in Table 4. According to the negative ΔG^o^ values obtained for both adsorbents, uptake of BB is spontaneous in nature^29^. This corroborates the favourable adsorption deduced from the Langmuir isotherm separation factor R_L_. Also, there was an increase in the negativity of ΔG° for both STpe and STpe-AgNP when solution temperature increased from 300 to 323 K, indicating that higher temperature enhances BB uptake onto the adsorbents^86^. Increasing disorderliness at the solution-adsorbent interface during sequestration of BB onto STpe and STpe-AgNP was inferred by positive ΔS° entropy values^87^. The increase in removal of BB with increasing temperature discussed previously was supported by the positive ΔH° values found, which are ascribed to an endothermic adsorption. Generally, positive ΔH^o^ values in the range of 2.1–20.9 kJ/mol signify a physisorption process, while ΔH° in the range of 20–200 kJ/mol is attributed to chemisorption^88^. In this study, ΔH° values of 16.77 kJ/mol and 15.70 kJ/mol were obtained for STpe and STpe-AgNP respectively, which indicated that the adsorption of BB dye is dominated by physisorption. This physisorption mechanism is in line with the suggestions of both the isotherm and kinetic analyses discussed previously. The advantage of physisorption is that it involves weaker binding of the adsorbate to the adsorbent, which enables easy regeneration and reusability of the adsorbent if required^33^.

**Table 4:** Thermodynamic parameters of bromophenol blue adsorption onto adsorbents STpe and the STpe-AgNP hybrid.

Adsorbent	T (K)	K_c_	ΔG° (kJ/mol)	ΔH° (kJ/mol)	ΔS° (J/mol K)	R^2^
STpe	300	1.78	−1.44	16.77	60.25	0.9492
	306	1.80	−1.50			
	313	2.21	−2.06			
	318	2.47	−2.40			
	323	2.79	−2.75			
STpe-AgNP	300	2.33	−2.11	15.70	59.33	0.9618
	306	2.61	−2.44			
	313	2.91	−2.78			
	318	3.50	−3.32			
	323	3.55	−3.40			

### Dye adsorption mechanism

**Figure 6:**
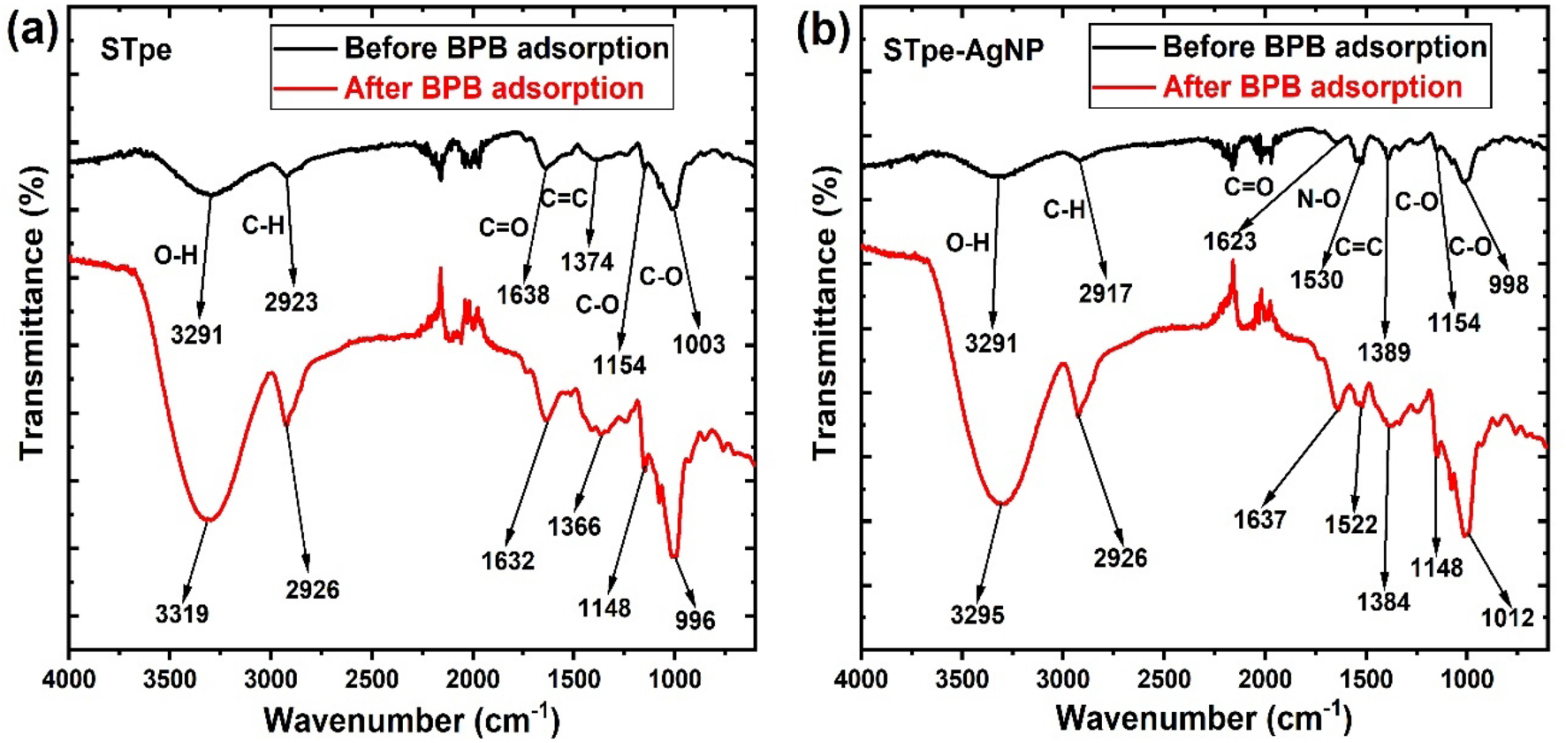
Fourier transform infrared spectra of (**a**) STpe and (**b**) STpe-AgNP before and after bromophenol blue dye adsorption.

The FTIR spectra of STpe and STpe-AgNP based on changes in the absorption bands was used to evaluate the mechanism of adsorption of BB dye as shown in Fig. 6. For the pristine STpe, shifts in the absorption bands were observed, from 3291 to 3319 cm^−1^ (OH), 2923 to 2926 cm^−1^ (C–H), 1638 to 1632 cm^−1^ (C=O), 1374 to 1366 cm^−1^ (C=C), 1154 to 1148 cm^−1^ (C–O) and 1003 to 996 cm^−1^ (C–O). Similarly, STpe-AgNP hybrid showed band shifts, from 3291 to 3295 cm^−1^ (OH), 2917 to 2926 cm^−1^ (C–H), 1623 to 1637 cm^−1^ (C=O), 1530 to 1522 cm^−1^ (N–O), 1389 to 1384 cm^−1^ (C=C), 1154 to 1148 cm^−1^ (C–O) and 998 to 1012 cm^−1^ (C–O). The shifts in several absorption bands after the adsorption process indicates the utilization of the associated functional groups for BB dye adsorption on both STpe and STpe-AgNP. Therefore, the impregnation of AgNPs on the hybrid did not alter the functional interaction with BB molecules in solution. In addition, the active involvement of the OH, C–H, C=O, C=C and C–O groups in the adsorption process shows that the mechanism of BB uptake on the materials was based on electrostatic, H-bonding, hydrophobic and π–π interactions^89^. Similar mechanistic interaction was also obtained in the adsorption of BB dye onto magnetic nanoparticle-*Musa acuminata* peel composite^25^.

### Effect of interfering ions on BB removal

**Figure 7:**
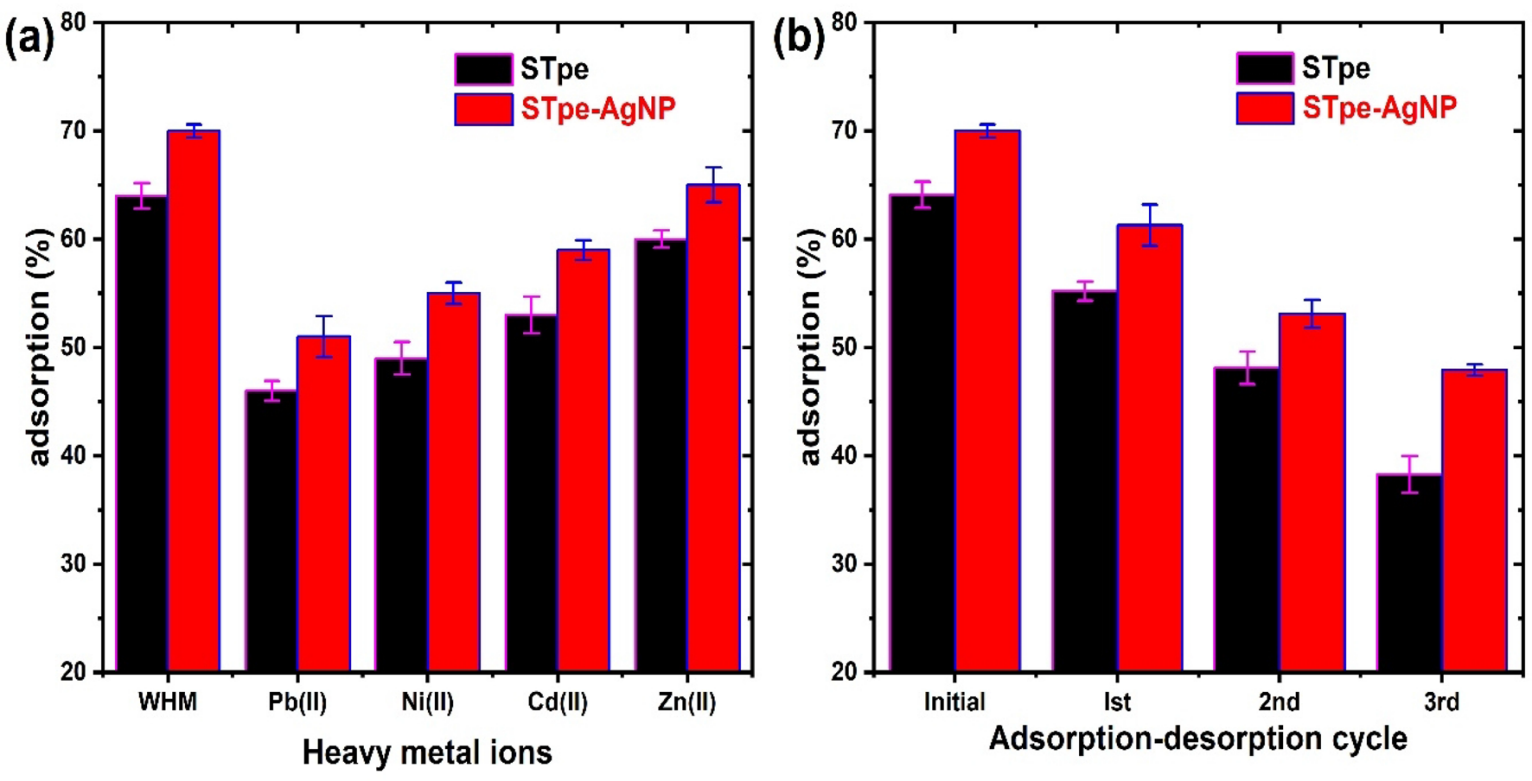
(**a**) Effect of interfering metal ions on the adsorption of BB dye by the two adsorbents (where WHM = without heavy metal), and (**b**) efficiency of BB adsorption onto the two adsorbents after each adsorption–desorption cycle, using 0.5 M NaOH solution as eluent for desorption.

Industrial wastewaters from industries are usually contaminated with heavy metals along with dyes. It is therefore important to determine the adsorption behaviour of BB dye in the presence of other common heavy metal pollutants in solution. The influence of the presence of Pb(II), Ni(II), Cd(II) and Zn(II) metal ions on the adsorption of BB onto biosorbents STpe and STpe-AgNP, was thus evaluated. We prepared various solutions containing 50 mg/L of BB dye along with 50 mg/L of each heavy metal concentration, in four separate 100 mL volumetric flasks. This ensured that the dye and heavy metals were present in the same concentration. The respective adsorbents (0.06 g) were then contacted with 10 mL of a given bi-component solution at pH 4.0, and ultra-sonicated for 120 min at a temperature of 300 K. Each mixture was then centrifuged and the filtrate analyzed for residual BB concentration. The result of the effect of the interfering metal ions is shown in Fig. 7a. WHM (without heavy metal) signifies the adsorption of BB in the absence of metal ions in solution. We observed that heavy metal presence in solution slightly decreased the uptake of BB onto both STpe and STpe-AgNP. The decrease was more significant in the presence of Pb(II) ions, with BB adsorption decreasing from 64 to 46% for STpe, and from 68 to 51% for the STpe-AgNP hybrid. The decrease of BB adsorption in the presence of heavy metals could be attributed to the occupation of some of the adsorbent active sites by metal ions. However, the results showed that BB is still very much adsorbed onto both adsorbents, even in the presence of heavy metals. The pH of 4.0 must have favored BB uptake above the heavy metals, as the surface of the adsorbents is still positively charged at this pH, thus repelling the positively charged metallic cations and rather attracting the anionic dye molecules. The heavy metals in solution hindered the adsorption of BB in decreasing order of Pb(II) > Ni(II) > Cd(II) > Zn(II). This trend is strongly related to their decreasing electronegativity of Pb (2.33) > Ni (1.91) > Cd (1.69) > Zn (1.65) and increasing hydrated ionic radius of Pb (0.401 nm) < Ni (0.404 nm) < Cd (0.426) < Zn (0.43 nm) of the heavy metal ions^25^. Thus, the higher the electronegativity, the greater the affinity of metal ions for the adsorbent active sites, and the lower the uptake for BB. This is attributed to greater competition between the more electronegative heavy metals and BB for the active sites of the adsorbents. On the other hand, the smaller hydrated radius creates easier diffusion of heavy metal ions, for efficient interaction with adsorbent functional groups^90^. Therefore, these more favoured metal ions are adsorbed, which decreases the remaining actives sites available for the BB dye binding onto STpe and STpe-AgNP. Importantly, the hybrid adsorbent impregnated with AgNPs showed higher adsorption than pristine STpe in the presence of all heavy metals studied. These results also suggest that the adsorbents from this study could be efficient for the decontamination of heavy metals from water at higher pH values > 6.0, where the adsorbents’ surfaces become negatively charged.

### Regeneration and reusability of STpe and STpe-AgNP

Apart from the efficient adsorption potential of an adsorbent, the regeneration and reusability of the material is also important, to avoid secondary pollution from the adsorbate-loaded adsorbent when disposed to the environment. We thus evaluated the regeneration of STpe and STpe-AgNP using 0.5 M NaOH as eluent, as well as its reusability for BB adsorption, within three adsorption–desorption cycles. BB desorption of 91.3% and 88.5% was obtained from the dye-loaded STpe and STpe-AgNP, respectively. This recorded high desorption corroborates the physical adsorption of BB^27^. Since high pH values in the alkaline region were found to be unfavorable for BB adsorption, utilizing the alkaline NaOH solution as eluent, enhanced desorption of BB from the surface of the materials. The reusability of the adsorbents within three adsorption–desorption cycles is illustrated in Fig. 7b. As observed, there was a steady decrease in the percentage adsorption of bromophenol blue, decreasing from 64.1% (first adsorption) to 38.3% onto the STpe biosorbent, and from 68.3% (first adsorption) to 47.6% onto the STpe-AgNP hybrid, after three cycles. This decrease can be ascribed to weight loss of the material, functional group loss and incomplete desorption of BB from the adsorbents during recycling^91^. Similar results were observed in the adsorption–desorption of phosphate by AgNP impregnated activated carbon derived from tea residue^53^. The results obtained revealed that STpe and STpe-AgNP can be used as efficient adsorbents for BB within limited cycles of reuse. However, since the adsorbents are readily available as waste biomass and involve simple preparation procedures, they can easily and cheaply be replaced. The spent biosorbents could be utilized as a source of biofuels, in landfills and as additives in the production of building blocks and firebricks^92,93^.

## Conclusions

Pristine *Solanum tuberosum* peel (STpe) was successfully impregnated with Ag nanoparticles to form a hybrid adsorbent (STpe-AgNP), which was applied for the abstraction of bromophenol blue from water. The Fourier transform infrared spectra showed various functional groups on the hybrid adsorbent’s surface for slightly more effective adsorption of bromophenol blue. Thermo-gravimetric analysis showed that the thermal stability of the hybrid was enhanced by the impregnation of AgNP, when compared to the pristine adsorbent. An increase in percentage uptake of bromophenol blue was obtained with increase in adsorbent dose and sonication time, after which uptake became constant. On the other hand, a decrease in adsorption with increasing pH and bromophenol blue concentration was observed. The AgNP impregnated hybrid was found to be water stable at various pH values of 2.0–9.0. Both the pristine and hybrid adsorbents were heterogeneous in nature, as was deduced from the best fit of the Freundlich isotherm, which was verified by the independent oriented site analysis. The kinetic analysis was best fitted by the intraparticle diffusion and pseudo-first order models. Endothermic and spontaneous adsorption of bromophenol blue was deduced from thermodynamic analysis. The presence of interfering metal ions in the simulated wastewater reduced the adsorption of bromophenol blue in the order Pb(II) > Ni(II) > Cd(II) > Zn(II), with the least interference from Zn(II) ions. AgNP impregnation of pristine *Solanum tuberosum* peel was found to slightly increase the maximum uptake capacity from 8.157 to 9.604 mg/g. The mechanism of bromophenol blue adsorption onto the pristine STpe and STpe-AgNP hybrid was found to be based on H-bonding, hydrophobic, electrostatic and π–π interactions. Efficient desorption of bromophenol blue greater than 88% from both adsorbents was achieved using 0.5 M NaOH solution as eluent, although both adsorbents exhibited limited reusability. The pure biosorbent *Solanum tuberosum* (potato) peel (STpe) performed only slightly worse than the chemically modified *Solanum tuberosum* peel**–**silver nanoparticle hybrid (STpe-AgNP). However, since low-cost water purification methods are urgently needed in developing countries, the pure biosorbent STpe that is readily available worldwide as biomass waste, is a viable option to be used for the removal of bromophenol blue from wastewater. The spent adsorbents could be reutilized as a source of biofuels, in landfills, and as additives in the production of building blocks and firebricks.

## Abbreviations

BB: Bromophenol blue
STpe: *Solanum tuberosum* Peel
STpe-AgNP: *Solanum tuberosum* Peel**–**silver nanoparticle hybrid
FTIR: Fourier transform infrared
pHpzc: PH point of zero charge
XRD: X-ray diffraction
FWHM: Full width of the half-maximum diffraction peak
SEM: Field emission scanning electron microscopy
EDX: Energy dispersive X-ray
TGA: Thermo-gravimetric analysis
BET: Brunauer–Emmett–Teller
S_BET_: Brunauer–Emmett–Teller surface area
PFO: Pseudo-first order
PSO: Pseudo-second order
LFD: Liquid film diffusion
ID: Intraparticle diffusion
WHM: Without heavy metal
